# Understanding Lay Counselor Perspectives on Mobile Phone Supervision in Kenya: Qualitative Study

**DOI:** 10.2196/38822

**Published:** 2023-02-02

**Authors:** Noah S Triplett, Clara Johnson, Sharon Kiche, Kara Dastrup, Julie Nguyen, Alayna Daniels, Anne Mbwayo, Cyrilla Amanya, Sean Munson, Pamela Y Collins, Bryan J Weiner, Shannon Dorsey

**Affiliations:** 1 Department of Psychology University of Washington Seattle, WA United States; 2 Department of Psychiatry University of Nairobi Nairobi Kenya; 3 Research Department Ace Africa Bungoma Kenya; 4 Human Centered Design & Engineering University of Washington Seattle, WA United States; 5 Department of Psychiatry and Behavioral Sciences University of Washington Seattle, WA United States; 6 Department of Global Health University of Washington Seattle, WA United States; 7 Department of of Health Services University of Washington Seattle, WA United States

**Keywords:** task shifting, human-centered design, supervision, global mental health, acceptability, feasibility, mobile phone

## Abstract

**Background:**

Task shifting is an effective model for increasing access to mental health treatment via lay counselors with less specialized training that deliver care under supervision. Mobile phones may present a low-technology opportunity to replace or decrease reliance on in-person supervision in task shifting, but important technical and contextual limitations must be examined and considered.

**Objective:**

Guided by human-centered design methods, we aimed to understand how mobile phones are currently used when supervising lay counselors, determine the acceptability and feasibility of mobile phone supervision, and generate solutions to improve mobile phone supervision.

**Methods:**

Participants were recruited from a large hybrid effectiveness implementation study in western Kenya wherein teachers and community health volunteers were trained to provide trauma-focused cognitive behavioral therapy. Lay counselors (n=24) and supervisors (n=3) participated in semistructured interviews in the language of the participants’ choosing (ie, English or Kiswahili). Lay counselor participants were stratified by supervisor-rated frequency of mobile phone use such that interviews included high-frequency, average-frequency, and low-frequency phone users in equal parts. Supervisors rated lay counselors on frequency of phone contact (ie, calls and SMS text messages) relative to their peers. The interviews were transcribed, translated when needed, and analyzed using thematic analysis.

**Results:**

Participants described a range of mobile phone uses, including providing clinical updates, scheduling and coordinating supervision and clinical groups, and supporting research procedures. Participants liked how mobile phones decreased burden, facilitated access to clinical and personal support, and enabled greater independence of lay counselors. Participants disliked how mobile phones limited information transmission and relationship building between supervisors and lay counselors. Mobile phone supervision was facilitated by access to working smartphones, ease and convenience of mobile phone supervision, mobile phone literacy, and positive supervisor-counselor relationships. Limited resources, technical difficulties, communication challenges, and limitations on which activities can be effectively performed via mobile phone were barriers to mobile phone supervision. Lay counselors and supervisors generated 27 distinct solutions to increase the acceptability and feasibility of mobile phone supervision. Strategies ranged in terms of the resources required and included providing phones and airtime to support supervision, identifying quiet and private places to hold mobile phone supervision, and delineating processes for requesting in-person support.

**Conclusions:**

Lay counselors and supervisors use mobile phones in a variety of ways; however, there are distinct challenges to their use that must be addressed to optimize acceptability, feasibility, and usability. Researchers should consider limitations to implementing digital health tools and design solutions alongside end users to optimize the use of these tools.

**International Registered Report Identifier (IRRID):**

RR2-10.1186/s43058-020-00102-9

## Introduction

### Background

Most individuals worldwide cannot access needed mental health treatments. Task shifting has emerged as an effective and potentially sustainable solution for addressing the human resource shortages that contribute to the mental health treatment gap [1]. In task shifting, less specialized providers, including lay providers (eg, community health workers and teachers) without formal mental health training or experience, are trained to deliver evidence-based psychotherapies (EBPs) under supervision [1,2]. Although evidence continues to support the effectiveness of task shifting, further research is needed to understand how to scale up and sustain task shifting, including how to sustain supervision [3,4].

Research in high-income settings suggests that ongoing supervision is necessary to ensure that EBPs are delivered with fidelity (ie, as intended by intervention developers [5]). Further ongoing supervision and support may be important to ensure patient safety and, for lay counselors in particular, to protect against burnout by providing support with the demands and challenges of taking on additional roles and responsibilities [6]. However, the costs of ongoing supervision have been a challenge for EBP delivery across community settings in the United States [7,8]. Challenges with in-person supervision are amplified in low-resource contexts such as low- to middle-income countries (LMICs), where funding is low and trained mental health providers who can serve as supervisors are more limited. To address some of these challenges in LMICs, communities and researchers have successfully examined the utility of group supervision and supervision led by community members [9-11]. Nonetheless, these solutions do not fully address the barriers related to the cost and inconvenience of in-person supervision in low-resource contexts.

There may be opportunities to leverage digital technology as a tool to supervise lay counselors and to decrease the need for in-person supervision**.** As access to and use of mobile phones continue to rise worldwide [12], there has been increased research examining the use of digital technology and cellphones in LMICs [13]. Most of this research has come from the field of information and communication technology and development and has documented the benefits and challenges of mobile phone use in LMICs, such as challenges with network connection and sharing mobile phones among multiple individuals [14-16]. Research has also noted the importance of considering the limitations of mobile phones when designing and implementing digital technologies in LMICs [17]. Similar benefits and limitations emerge from research focused on the use of digital support for health care provision in LMICs [18,19]; however, less work has examined how mobile phones can be used as tools to support task shifting of mental health treatment, which may require more tailored support than other types of health care provision (eg, role-playing how to discuss a traumatic event).

Psychology and global mental health researchers have called for studies evaluating the potential of digital health tools to improve mental health services [20-22]. In LMICs specifically, an emerging body of literature has examined the use of digital health tools to support lay counselors, including facilitating training [23,24], providing tools for diagnosis [25,26], and supporting supervision [19,27-29]. However, although this work may use digital health solutions, it has not explicitly discussed the various ways in which mobile phones are used to support service provision, nor has it focused on the support needed to facilitate digital health solutions. Existing research has largely implemented digital tools without reporting on lay providers’ perceptions of those tools or their suggested support to facilitate the implementation of digital health tools. Research that has focused on providers’ experiences [19] has not been related to task-shifting mental health care, which may have unique considerations. Considering the challenges and preferences of those likely to use mobile phones for supervision is essential to ensure that mobile phones can be used effectively. Without considering these contextual factors, a push for greater mobile phone supervision (or other digital health tools) may create additional gaps between those who can and cannot use the technology, thereby inadvertently contributing to inequities.

To maximize the acceptability (ie, satisfaction; [30]) and feasibility (ie, utility or practicability; [30]) of using mobile phones to supervise lay counselors, their implementation must be guided by the needs and preferences of supervisors and lay counselors. Human-centered design (HCD) offers a guiding framework for implementation researchers to understand and incorporate the needs and preferences of end users [31], including in global health research [32] and mental health intervention and implementation support development [33,34]. By understanding and addressing user preferences at each step of implementation, the acceptability, sustainability, and equity of such interventions and support may be increased.

### Objectives

This manuscript presents the first phase of an HCD research project intended to investigate how mobile phones can be leveraged as digital health tools for the supervision of lay counselors in western Kenya, including potentially replacing in-person supervision. We examined how mobile phones are used for supervision within a National Institute of Mental Health–funded cluster randomized controlled trial, “Building and Sustaining Interventions for Children (BASIC): Task Sharing Mental Health Care in Low Resource Settings” [35]. The BASIC trial began in 2018 and, at the time of this study, had trained 210 lay counselors (105 teacher counselors and 105 community health volunteer [CHV] counselors) to deliver a group-based and culturally adapted trauma-focused cognitive behavioral therapy (TF-CBT; [36]). Lay counselors work together in groups of 3 to provide the treatment and are trained and supervised by Kenyan supervisors who are experienced lay counselors previously trained and supervised in TF-CBT as part of a randomized controlled trial [37] that preceded BASIC. To add to the literature on how mobile phones are currently used and provide context for design, we used qualitative interviews to explore the current context of mobile phone use and the acceptability and feasibility of scaling up mobile phone supervision. In line with HCD, we also sought to co-design solutions to improve mobile phone supervision by eliciting strategies from lay counselors and supervisors (ie, end users) on how to improve mobile phone supervision in their context. Both aims add the voices of lay mental health counselors and supervisors themselves to the literature—a major point of novelty. By working alongside lay counselors and supervisors to inform and improve the implementation of mobile phone supervision, we aimed to overcome potential challenges and improve the sustainability of task shifting in lower-resource settings. By conducting this research with lay counselors in western Kenya, we hope to generate techniques that a range of low-resource communities worldwide can apply to better leverage mobile phones as a low-technology digital health tool.

## Methods

### Overview

This study was conducted as part of a larger study examining the use of mobile phones to supplement or replace in-person supervision with lay counselors in western Kenya [38]. This trial builds on a National Institute of Mental Health–funded cluster randomized controlled trial, “Building and Sustaining Interventions for Children (BASIC): Task Sharing Mental Health Care in Low Resource Settings” [35]. BASIC represents a long-standing collaboration between researchers in the United States and Kenyan partners at Ace Africa. As stated previously, the BASIC trial has trained 210 lay counselors, which includes both 105 teacher counselors from schools in western Kenya and 105 CHV counselors who work within the health sector. Lay counselors work together in groups of 3 to deliver a group-based and culturally adapted TF-CBT [36]. Lay counselor groups are based on treatment delivery location, and the same groups of lay counselors work together (ie, groupings are not varied) except when teachers or CHVs move and replacement counselors are identified. Lay counselors are supervised by Kenyan supervisors who are full-time employees at Ace Africa and exclusively provide supervision and training (ie, they themselves do not lead treatment groups). All supervisors were previous lay counselors who gained experience delivering the intervention as part of a randomized controlled trial [37] that preceded BASIC.

Supervisors meet in person with each group of lay counselors after training (to practice the intervention and prepare for delivery) and at least four times during their first round of implementing groups. In-person supervision was provided in a group format. In addition to routine administrative supervision, in-person meetings consisted of reviewing past sessions and practicing (role-plays) for upcoming sessions, discussion of any challenging treatment elements, and problem-solving of any unique children and guardian participant needs. Supervision included ad hoc mobile phone communications via SMS text messages and phone calls during times between in-person meetings. Mobile phone communication included both individual and group SMS text messages and calls. Mobile phones were not provided by the BASIC trial, and all lay counselors used their own phones. The COVID-19 pandemic increased the team’s reliance on mobile phones to provide support. Despite the reliance on mobile phones to provide support from a distance, the extent to which mobile phones can be used and systematically implemented to support supervision remains understudied. These results are from the first aim of a pilot trial intended to understand the barriers to and facilitators of mobile phone supervision and co-design and test strategies to optimize its acceptability, feasibility, and usability. See Triplett et al [38] for the procedures and methods of the larger study.

### Participants

To understand how mobile phones were used, interviews were conducted with supervisors and lay counselors who received training in (and subsequently delivered) TF-CBT as part of the BASIC trial. Lay counselor participants were selected via a stratified random sampling approach to balance participants across counselor types (ie, teacher and CHV counselors) as well as those who used mobile phones with varying frequencies. Supervisors categorized 180 lay counselors who had completed TF-CBT delivery into one of three categories: (1) high-frequency users, (2) average-frequency users, and (3) low-frequency users. Supervisors rated all the lay counselors they directly supervised based on frequency of all types of phone contact (ie, phone calls; SMS text messages; and WhatsApp messages, if applicable). Supervisors rated lay counselors relative to the average peer (eg, high-frequency users were those perceived as having a higher-than-average number of contacts). Interviewing “extreme” users—those using mobile phones with high frequency or rarely—is an HCD technique that is intended to more easily illustrate the range of behaviors and needs of a population [39]. Per the published protocol [38], we planned for supervisors to rate counselors’ use on a 1 to 7 Likert-type scale; however, a narrower rating scale was ultimately more feasible. After all counselors were rated for mobile phone use frequency, a US-based team member stratified participants by counselor type (teacher or CHV) and frequency of mobile phone use. A random number generator was used to randomly select participants. We selected 12 participants from each lay counselor type (ie, 12 CHVs and 12 teachers) as 12 is generally considered sufficient for thematic saturation [40]. Mobile phone use was balanced across counselor types (ie, 8/24, 33% of the total sample was from each use category, and 12/24, 50% of those users were teachers and CHV counselors). The supervisor participants were the 3 supervisors who remained employed with our Kenyan partner, Ace Africa, at the time of this study. These supervisors were the only current supervisors in the parent study. Given that supervisors were all active and frequently connected with their lay counselors, we did not rate their frequency of use. It was assumed that all supervisors would have been considered high-frequency users. Interviewers approached participants via telephone to invite them to take part and gather informed consent. There were no exclusion criteria for lay counselors or supervisors.

All those invited agreed to participate in the interviews, and for counselors, interviews were conducted where they delivered the treatment. Lay counselor participants were 33% (8/24) men, as were the supervisor participants (1/3, 33%). Owing to a recruitment error, one-third of the participants (8/24, 33%) used their mobile phones with high frequency, and slightly different numbers used their phones with average frequency (9/24, 38%) and low frequency (7/24, 29%). Of the 24 lay counselor participants, 6 (25%) did not have smartphones. These participants were all CHVs and were split across high-frequency (2/6, 33%) and low-frequency (4/6, 67%) users. No other nonparticipants were present in the interviews.

### Procedures

Semistructured interviews were conducted by a trained study interviewer in the language of the participants’ choosing (ie, Kiswahili or English). Although other languages were spoken in the study catchment area, all research activities from the parent trial were conducted in English and Kiswahili as community members indicated a preference for these 2 languages. As such, we opted to conduct interviews in these preferred and official languages. Code switching, or alternating between languages, was observed in some interviews. Participants were free to switch between English and Swahili, although interviewers directed them back to those languages when they spoke other, nonstudy languages (eg, Luhya). Interviewers were both male and female and had at least an undergraduate degree. Interviewers had completed all study interviews for the parent trial and already knew the participants. Each interview lasted approximately 1 hour. No repeated interviews were conducted. Supervisor interviews were completed by the first author, a White male graduate student from the United States. All supervisor interviews were conducted in English.

Interview protocols began broadly, first reminding interviewees of the goals of the study and then asking lay counselors and supervisors to reflect on how they used their mobile phones to communicate regarding treatment delivery in their respective roles. Questions then became more tailored to examine what they liked most about using mobile phones for supervision, the challenges or frustrations with mobile phone supervision, and the degree to which they felt that mobile phones could replace in-person supervision. Drawing from HCD techniques, the final question asked participants to describe how they would use their mobile phones during a specific “scenario of use” [41], a hypothetical scenario in which they were preparing for a treatment group and needed to request supervision via their mobile phones. The interview guide was piloted and refined with support from study interviewers. The final interview guide is provided in Multimedia Appendix 1. The interviews were recorded with participant permission, transcribed, and translated (if applicable). Interviewers also took notes during the interviews to ensure that participant responses were comprehensive and note any places that may need additional clarification before concluding the interviews. Transcripts were not distributed to participants for review, but selected transcripts were reviewed in conjunction with audio by members of the research team.

### Analysis

Interview transcripts were coded and analyzed following the 6-phase framework by Braun and Clarke [42] for thematic analysis. Transcripts were coded in Dedoose (SocioCultural Research Consultants) by researchers in the United States. Kiswahili interviews were translated by native Kiswahili speakers and trained translators. The lead author, NST, along with 2 other authors, SK and CJ, initially reviewed a random sample of 6 interviews, independently generated codes, and collaboratively developed an initial codebook. The remaining interviews were assigned to pairs of coders who coded independently and then met to discuss all codes to reach a consensus. A third coder was involved to resolve any discrepancies. In total, 3 additional coders who were not engaged in codebook development supported qualitative coding, for a total of 6 coders. All coders had experience conducting qualitative research, and the lead author led all coding and training for the coders. Most coders (5/6, 83%) were United States born and fluent only in English, and 1 author, SK, was born in Kenya and was fluent in both English and Kiswahili. Each coding pair included 1 member of the original codebook team.

The codebook was considered a “live document” and iteratively refined throughout the coding process. After completing all coding, the results were presented back to the interview participants for member checking at an in-person workshop. This workshop also included other HCD activities, which have been reported elsewhere. Codebook definitions were refined during the workshop; however, no new themes emerged. The codebook team (NST, CJ, and SK) worked together to propose and refine qualitative themes that grouped together the member-checked codes. Qualitative methods and results are presented in concordance with the COREQ (Consolidated Criteria for Reporting Qualitative Research [43]) guidelines, and the COREQ checklist is available in Multimedia Appendix 2.

### Ethics Approval

The institutional review boards at the University of Washington and Kenya Medical Research Institute approved all study procedures (STUDY00010734). The Kenya National Commission for Science, Technology, and Innovation also reviewed and approved this research (NACOSTI/P/16/28122/14518). Informed consent was obtained from all participants before the interviews were conducted. All the interview excerpts were deidentified. Participants received a small incentive for taking part (equivalent to US $5).

## Results

### Overview

In total, 24 lay providers (n=12, 50% teacher counselors and n=12, 50% CHV counselors) and 3 supervisors were invited and interviewed in June 2021. Of the 24 lay counselor participants, 6 (25%) did not have a smartphone. These participants were all CHVs and were split across high-frequency (2/6, 33%) and low-frequency (4/6, 67%) users. The complete demographics are presented in Table 1. Multiple themes emerged from the interviews, including information on the various uses of mobile phones to support delivery, likes and dislikes of mobile phone supervision, barriers to and facilitators of using mobile phones for supervision, and strategies to overcome barriers. Direct quotes or translations are presented to contextualize the themes.

**Table 1:** Demographic and baseline characteristics (N=25).

Characteristic			Teacher counselors (n=10)^a^		CHV^b^ counselors (n=12)		Supervisors (n=3)
**Sex, n (%)**							
	Male	4 (40)		3 (25)		1 (33)	
	Female	6 (60)		9 (75)		2 (67)	
**Smartphone, n (%)**							
	No	0 (0)		6 (50)		0 (0)	
	Yes	10 (100)		6 (50)		3 (100)	
**Received previous training in psychosocial counseling, n (%)**							
	No	5 (50)		4 (33)		0 (0)	
	Yes	5 (50)		8 (67)		3 (100)	
**Provided previous psychosocial counseling, n (%)**							
	No	1 (10)		5 (42)		0 (0)	
	Yes	9 (90)		7 (58)		3 (100)	
Age (years), mean (SD)			42.7 (8.2)		50.8 (12.0)		36.3 (7.8)

^a^Demographic data were missing for 2 teacher counselors.

^b^CHV: community health volunteer.

### Use of Mobile Phones

Lay counselors and supervisors described three subthemes of mobile phone uses to support intervention delivery: (1) requesting and providing advice and updates on clinical content, (2) requesting and scheduling in-person supervision, and (3) requesting and providing advice on research procedures.

#### Advice and Updates on Clinical Content

Participants described requesting and providing advice on clinical content as well as providing routine clinical updates on clients’ symptoms via mobile phone. When supervisors were unable to provide face-to-face supervision, lay counselors received advice on how to deliver clinical content via phone calls or SMS text messages. Supervisors provided clarifications on clinical content and supported lay counselors when they encountered specific clinical challenges. A lay counselor explained the following:

Maybe I’m doubting something in the session I’m going to present, so I want some clarification about what I’m going to do...Then maybe [my supervisor] will tell me [what to do]...you find that I have solved that problem.

Another counselor discussed the benefits of mobile phones for providing support with clinical issues that arose during sessions:

So I had [a] problem and then in the middle of the session I had to call. When I called, [they] responded very quickly, [they] told me [how to proceed]...so it helped me very much...it was a quick response and it helped me in the middle of the session.

#### Scheduling and Coordinating

Participants emphasized the value of mobile phones in scheduling and coordinating clinical activities, including clinical practice, sessions, and supervision. Following their in-person clinical training, supervisors “communicated to [lay counselors] on when to start the [intervention] through the phone and...instructed [them] on how [they] should schedule [their] lessons with clients...with the phone, [lay counselors] were able to mobilize clients and started the [intervention].” Lay counselors also used mobile phones to connect and coordinate with their cocounselors, such as planning practice or coordinating clinical duties:

Sometimes we call each other. I can call or text them. But most of all I just call them on the phone.

Beyond scheduling clinical sessions, mobile phones were instrumental in scheduling supervision—both in person and via phone. A counselor explained the importance of communicating before in-person supervision to schedule and coordinate:

If I’m informed on time, maybe I will try to plan as per...I will try maybe telling the supervisor that, “At this time, I will not be available, or I will be available.” If I’m informed on time, I will try to avail myself rather than being ambushed...

Scheduling and coordinating were similarly important for phone calling:

I could just SMS and say it is heavily raining here, maybe we’ll meet later. And then [my supervisor] will respond it’s okay.

#### Research Procedures

A final key use of mobile phones was communicating with supervisors regarding the research procedures for the parent BASIC trial. Supervisors reminded lay counselors about reporting requirements and advised them on issues related to participant recruitment and attendance. Lay counselors photographed and sent anonymized research forms to their supervisors via SMS text message or WhatsApp; they would also request paper copies of forms or other intervention materials via phone. A lay counselor stated the following:

You find that it easy even to send a report through the phone. Because you’re just preparing a report and then if it is taking a photograph and then I’ll send it through WhatsApp...

Finally, lay counselors were provided with airtime or other incentives for participating in the research trial via their mobile phones and mobile money.

### Acceptability

#### Overview

Lay counselors and supervisors described both likes and dislikes associated with using their mobile phones to support treatment delivery. Likes were categorized into three subthemes: (1) decreased lay counselor and supervisor burden, (2) facilitated clinical and personal support, and (3) increased independence. Dislikes were also categorized into two subthemes: (1) limited information transmission and (2) affected ability to build rapport. The differences in the percentage of interviews in each use category that mention each acceptability theme are presented in Multimedia Appendix 3.

#### Like: Decreasing Burden

Participants frequently described how using mobile phones for supervision decreased burden for the entire team—both lay counselors and supervisors. Using mobile phones for clinical supervision allowed counselors and supervisors to decrease travel required for in-person supervision, saving time and costs:

mobile communication [allows] the supervisor to reach interior places without a problem...During the rainy season, the supervisors get a very big problem in traveling. Therefore, when there is a mobile communication, the teacher and the supervisor will just communicate.

This was frequently associated with decreased risks of in-person supervision, both in terms of decreasing travel risks and protecting against the spread of COVID-19. A lay counselor stated the following:

The government is encouraging digital devices because the supervisor can travel from the office to school...Through the mobile phone they will only communicate, but the virus will not spread.

Before the adoption of mobile phones for communication, lay counselors and supervisors also communicated through written letters. Participants described the use of mobile phones as decreasing the burden of letter writing.

The aforementioned benefits of mobile phone supervision saved time for counselors and supervisors and resulted in lay counselors feeling that their counseling duties were more manageable. A counselor stated the following:

Once you call, you’re given a way forward, and you do it immediately without wasting time.

The time saving and convenience of mobile phone supervision also made providing supervision to lay counselors more feasible for supervisors. Counselors described this as something they liked about using mobile phones:

Personally, I will be satisfied [with mobile phone supervision]...Whatever I want, [they] can help me, even by phone...[they] will also be saving [their] time to serve us counselors because I know we are many. [They] will satisfy everyone.

#### Like: Facilitating Support for Counselors

Participants often discussed the benefits of mobile phones in facilitating support—both for clinical skills and personal well-being. Lay counselors were able to easily and frequently reach supervisors for support via mobile phone. A supervisor explained that they encouraged their lay counselors “if something pops in your head, or if you have a question, you can just text or just [call and hang up to avoid being charged], anytime*.*” Lay counselors described reaching out to supervisors for support with urgent matters, including challenges that arose during clinical sessions, and how receiving in-the-moment support enabled them to continue delivering content:

I had that problem and then in the middle of the session I had to call. When I called [they] responded very quickly...it helped me very much.

Lay counselors also appreciated the ease of access to support for nonurgent matters, which enabled them to obtain answers promptly without waiting for an in-person supervision meeting.

Participants also discussed the personal benefits of using mobile phones for supervision. Supervisors used mobile phones as tools to build morale and encourage lay counselors, often sending “good luck” or other inspirational messages to their lay counselors. A counselor described their feelings when receiving these messages:

[my supervisor was] encouraging me to do the job and also to encourage my colleagues to just work hard...I loved it so much, it made us work hard.

Mobile phone supervision was also a place for lay counselors to communicate with one another and get to know each other better:

As [lay counselors], we used the phone to get to know each other...

Finally, some lay counselors expressed an appreciation for the privacy and confidentiality afforded by mobile phone supervision. A supervisor described this as follows:

talking to them individually through phone, to me, it’s very helpful because they will be opening up, telling you how the session was. When you talk [to the group] everybody wants to be perfect, they don’t want to appear like they did something wrong...

#### Like: Increasing Independence

Finally, lay counselor participants specifically discussed how mobile phone supervision afforded them increased independence as counselors and, as a result, increased their confidence in their own abilities and teamwork. Mobile phone supervision indicated to lay counselors that supervisors trusted their ability to provide quality care:

You know sometimes when you leave people with freedom and trusting them, they even work better than just when you’re on their back...I feel good [as a result]...I feel trusted.

The independence afforded by mobile phone supervision also enabled lay counselors to trust each other more and develop more cohesive group dynamics:

phone communication made us trust each other and work without questioning each other.

#### Dislike: Limited Information Transmission

In addition to the many aspects of mobile phone supervision that the participants liked, certain dislikes arose from the qualitative interviews. Lay counselors disliked how communicating through phone with supervisors limited the transmission of information. Given that some lay counselors did not have smartphones, SMS text messages tended to be very brief. As such, phone calls allowed them to communicate in greater detail; however, brevity was also a challenge with calls, likely because of the pressure to reduce airtime use. A lay counselor stated the following:

You will briefly talk on [the] phone, but not about everything you need to know.

Lay counselors also disliked that receiving support over the phone did not lend itself well to demonstrations of clinical techniques. A counselor explained the following:

I want to believe that when it comes to those demonstrations, then face-to-face [supervision] cannot be replaced by mobile for clarity.

Mobile phone supervision also hindered lay counselors’ and supervisors’ ability to convey and examine body language and gestures. A supervisor noted the following:

The only problem [with mobile phone supervision] is that sometimes I can’t really observe the body language in terms of maybe the nonverbal gestures.

Lay counselors and supervisors stressed the importance of complete communication and examining body language with more complex treatment elements, such as completing children’s trauma narratives (ie, imaginal exposure).

#### Dislike: Affected Ability to Build Rapport

Lay counselor participants often highlighted the importance of establishing a strong relationship with supervisors and noted that mobile phone supervision limited their ability to establish a trusting relationship. A lay counselor explained that meeting face-to-face first is essential and then they “will have what it takes to express [themself] better than on the phone.” Another lay counselor agreed, saying that “face-to-face sometimes also enhances that particular...public rapport...between the supervisor,” which leads to closeness that will “also enhance or will encourage good relations.” Counselors also expressed concerns that increasing reliance on mobile phone supervision would result in fewer incentives from their supervisors, such as small meals or snacks during in-person supervision meetings.

### Feasibility

#### Overview

Facilitators were categorized into four subthemes: (1) access to working smartphones, (2) ease and convenience of smartphones, (3) cellphone literacy, and (4) a strong supervisor and counselor relationship. Barriers were also categorized into four subthemes: (1) limited resources and time, (2) technical difficulties, (3) communication challenges, and (4) contextual limitations on which activities can be effectively performed via mobile phone. The differences in the percentage of interviews in each use category that mention each feasibility theme are presented in Multimedia Appendix 3.

#### Facilitator: Access to Working Smartphones

Participants noted that having a working smartphone with access to reliable internet, cellular service, and electricity allowed lay counselors and supervisors to engage in mobile phone supervision. A lay counselor summarized it as follows:

if you get a good phone, which can access all those things, there is such a possibility that the work [supervision] should be done by phone without any doubt.

Another lay counselor mentioned that “if the network is available, we can communicate at all times.” Some lay counselors stated that access to alternative phones when personal phones were not available facilitated mobile phone supervision.

#### Facilitator: Ease and Convenience of Mobile Phone Supervision

In addition, lay counselors and supervisors described ways in which mobile phone supervision provides an easy and convenient alternative to in-person supervision. A lay counselor highlighted the following:

Anytime you call, [my supervisor’s] phone is always on. So, [they] have been a good supervisor because sometimes you can be in the middle of a session and maybe call and don’t get [them]...but...I’ve never called, and I missed my supervisor.

Decreased costs in terms of travel and time associated with mobile phone supervision also made supervision more feasible. The utility of mobile phone supervision in routine instances (eg, when a question arises during a session) also contributed to lay counselors reporting mobile phone supervision as feasible and easy to use. A lay counselor explained that, “in areas where things move on well without many hitches, then mobile supervision can work.”

#### Facilitator: Supervisor-Counselor Relationship

Participants reported that the trust and cooperation between supervisors and lay counselors contributed to the feasibility of mobile phone supervision. A lay counselor explained that “I think it’s just a matter of cooperation between the supervisor and the counselor...” A warm and supportive supervisory relationship may be particularly important in mobile phone supervision where visual cues may not be as clear (eg, body language and facial expressions). A lay counselor expanded on this idea:

Because without trust and cooperation I can send something [to my supervisor], I can send anything even if it is useless. It’s not good. But if we trust each other and work together here...the phone call is real.

#### Facilitator: Mobile Phone Literacy

A final important facilitator that counselors noted was familiarity with the platform (eg, WhatsApp or SMS text messages) used in mobile phone supervision:

after getting that information [about WhatsApp], then I can easily connect with the supervisor and communication takes place.

When counselors and supervisors are knowledgeable about the platform on which they are communicating, mobile phone supervision is possible. Supervisors and lay counselors noted how COVID-19 had increased mobile phone literacy, which had made mobile phone supervision easier to use in some instances.

#### Barrier: Limited Resources and Time

Participants noted a few tangible barriers to remote supervision, often discussing issues of phone airtime and the challenges of balancing competing priorities during phone calls. Participants repeatedly mentioned lack of airtime, which hindered their ability to connect with their supervisors:

Airtime. You may not have it. Maybe I have no money to buy it. And I want to talk to my supervisor. You see there is a problem.

Further challenging participants was the cost of owning a working smartphone, which some felt was needed to have the best experience with mobile phone supervision (ie, sending photos and videos and accessing WhatsApp). Finally, participants expressed challenges with balancing many competing priorities in their limited time. A participant said the following:

[our supervisor advised us that] we are going to have a session on mobile phone. Then during that time, we are doing an exam [with the children]...So, it becomes difficult to use that mobile phone at that time...

#### Barrier: Technical Difficulties

Another common barrier to remote supervision was related to physical issues with the phones themselves (eg, broken or with weak batteries) as well as problems with the network connection. Participants mentioned that keeping the phone charged was especially challenging when there was unreliable electricity and rolling blackouts. Lay counselors also had issues with the network where they were unable to place calls and other times where a poor network connection affected the quality of the phone call in such a way that they could not understand their supervisor clearly. All the aforementioned issues were complicated by weather, which affected both the power supply and network access. A participant explained the following:

Even if you can call someone on phone, then you are told that they are not available. Yet, their phone is on...the rain is coming this way...You find there is no network at my end.

#### Barrier: Communication Challenges

Remote supervision inherently affects the nature of communication between supervisor and counselor, and some of these changes were cited as barriers by the participants. The time delay between asking supervisors a question and receiving an answer also posed issues and increased the possibility of miscommunication. A participant noted the following:

Sometimes you could call the supervisor and then maybe [they’re] also engaged in a meeting, [they’ll] tell you, “I’ll call you later.” And sometimes [they] might call very late when that issue has been left or has been left unresolved like that, or you have solved within your knowledge.

Furthermore, participants experienced occasions in which phone conversations felt rushed or when they were interrupted or distracted because they did not have secure and confidential locations in which to conduct mobile phone supervision.

#### Barrier: Limitations on Which Activities Can Effectively Be Performed via Mobile Phone

The most cited barrier to remote supervision was that participants felt that there were some supervisory activities that could not occur over the phone. This code captured a range of challenges, from not being able to physically hand a supervisor a report or receive COVID-19 supplies (eg, hand sanitizer and masks) to concerns that lay counselors may not be taken seriously by management unless supervisors were seen in person. In addition, some participants said that remote supervision takes some of the responsibilities off the supervisor and puts them on the lay counselors themselves, such as conducting treatment sensitization with the administration and guardians or terminating treatment groups. Finally, many participants noted concerns that, without in-person supervision, other counselors may cut corners and not do their job as thoroughly:

You can’t put a worker in a field and expect him or her (to) weed out everything without your supervision. He’ll just tell you he’s weeded. And if you go you get grass. He has not weeded out the dirt.

### Solutions

Lay counselors and supervisors offered 27 discrete solutions or suggestions to improve the acceptability and feasibility of mobile phone supervision. Providing airtime and phones were among the most mentioned strategies to improve the acceptability and feasibility of mobile phone supervision. Lay counselors searching for and identifying locations with optimal network connection to take phone calls for supervision was also frequently mentioned. A lay counselor stated the following:

We always just look for [network connection]. You can stand somewhere where it can come all, or maybe you’re sitting somewhere where that network is not there. So, it needs you to move so that you get it.

Another less frequently mentioned strategy was to provide training on mobile phones and apps such as WhatsApp to facilitate use for lay counselors who may lack knowledge of mobile phones. Some lay counselors described the clinical and personal benefits of learning new features on their mobile phones:

The phone was a tool that helped me a lot especially at that time I came to learn how to use WhatsApp...I found myself in a new world through that part of the WhatsApp group.

Each of the solutions, along with its definitions and the challenges it was intended to address, is presented in Textbox 1.

**Solutions to improve acceptability and feasibility by targeted barrier.**

**Limited resources and time**

- Lay counselors who are less familiar with phones and their functions can be provided with *phone training*.
- Lay counselors can *flash supervisors*, where they call and hang up after 1 ring to avoid being charged minutes. Supervisors then call back using their own minutes.
- Lay counselors can *“borrow” airtime* from phone providers in cases of emergencies when they do not have airtime.
- Task-shifting projects should *provide airtime* to lay counselors as an essential tool for their work.
- Task-shifting projects should *provide phones* to lay counselors as an essential tool for their work.

**Technical difficulties**

- Lay counselors should *ensure that their phones are charged* in advance of groups and known supervision contact.
- Lay counselors should *ensure that their phones are working* in advance of group therapy sessions and known supervision contact.
- Lay counselors should *establish a pattern of minimally using phones on days* of groups and known supervision contact to ensure that phones stay charged.
- Lay counselors should *identify locations with strong network coverage* near their delivery sites or homes.
- Lay counselors can *borrow phones* from cocounselors, colleagues, or friends if they encounter difficulties with their own phones.
- Lay counselors can *replace phone batteries* with borrowed or extra batteries.
- Lay counselors can request that *others send updates* on their behalf when facing communication challenges.
- Lay counselors should plan to *send communications early* to accommodate any delays.
- Lay counselors can *delay communications when needed* to conserve resources.
- Supervisors can *share information and advice from other sites* to motivate lay counselors or aid them in troubleshooting challenges.

**Communication challenges**

- Supervisors can *share information and advice from other sites* to motivate lay counselors or aid them in troubleshooting challenges.
- Lay counselors and supervisors can quickly *call to notify that important or urgent messages have been sent* to avoid messages being missed.
- Lay counselors and supervisors can *designate 1 counselor to relay all messages to cocounselors.*
- Lay counselors and supervisors can create *group messages* to send all communications to all counselors at once.
- Supervisors can ensure that they are sending *individual messages to each lay counselor.*
- Lay counselors should *inform supervisors of the time of the sessions* so that supervisors can be available to support if needed.
- Lay counselors can *call supervisors during sessions for “live” supervision.*
- Lay counselors and supervisors can *send messages during sessions* for support and updates.
- Lay counselors and supervisors can *send forms via picture messages* for guidance.
- Lay counselors and supervisors can *video call for modeling and guidance.*
- Lay counselors should briefly *clarify their identity and purpose for calling* when needing support.

**Limitations on which activities can be effectively performed via mobile phone**

- Supervisors should *begin by providing in-person supervision* to build confidence and rapport and handle challenges.
- Lay counselors should always feel empowered to*request in-person assistance.*

## Discussion

### Use, Acceptability, and Feasibility

Although lay counselors and supervisors reported some dislikes with mobile phone supervision, it was overall reported to be acceptable and feasible. Lay counselors and supervisors generated unique solutions to improve the acceptability and feasibility of mobile phone supervision that are currently being explored in a pilot trial [38]. Mobile phones are already often used to support health care providers in LMICs, and other mental health projects have examined how mobile phones can be used as a tool to support supervision [27-29]. However, our study is among the first to gather lay counselor perspectives and explicitly examine how use of mobile phones can be optimized as a low-technology digital health tool to support lay counselors and supervisors in supervision—a key solution to increasing access to mental health care and improving mental health equity worldwide. Although the high number of likes and facilitators reported for acceptability and feasibility show the promise of mobile phone supervision, it would be a mistake to interpret these results as indicating that mobile phone supervision can completely replace in-person supervision. Instead, lay counselors and supervisors stressed that mobile phone supervision was an important add-on tool that can address shortfalls with in-person supervision. This important finding from our co-design process resulted in changing our research methods and question from focusing on completely replacing in-person supervision with mobile phone supervision to instead focusing on optimizing the use of mobile phones to supplement in-person supervision.

Most research on clinical supervision has been conducted with US graduate students or community mental health providers, the latter of which has shown that clinical supervision largely focuses on case management [44]. There has been limited work characterizing the supervision of lay counselors, particularly by other lay counselors and via mobile phone. Our work contributes important descriptive information about lay counselor supervision via mobile phone. It extends beyond clinical support to include personal support as well as additional ways to receive encouragement and motivation and foster self-confidence and team building. This is somewhat consistent with other research from Kenya on supervision as a protective factor against lay counselor burnout [6]. Our findings add nuance to the various ways in which supervision can protect against burnout via mobile phone, such as encouraging SMS text messages or reminders. Mobile phones enable lay counselors to feel supported from afar without the burden of traveling for supervision. Research from related fields has highlighted how phones can increase interpersonal connections in Kenya [15], although this work did not examine the intricacies of receiving clinical supervision via mobile phone. This finding is unique and important as we consider how to support a growing cadre of mental health care providers worldwide.

Although participants mentioned several facilitators, access to working smartphones was mentioned by almost every lay counselor participant (23/24, 96% of the total). Although access to and use of mobile phones continue to rise worldwide [12], there is still variability in who has access to working smartphones. Of the 24 lay counselor participants, 6 (25%) did not have a smartphone. Others explained that, although they had smartphones, their phones were broken or “fragile” (eg, having cracked screens or short battery lives). Extensive work has examined the challenges of mobile phone use in LMICs and how technology developers may “leave behind” key populations in designing digital tools [17], although these considerations have been largely absent from conversations regarding digital mental health solutions. Focusing solutions only on individuals who have working smartphones—individuals with relatively higher resources in these settings—would undoubtedly result in an inequitable digital health solution. Other task-shifting projects may consider providing smartphones for counselors or relying on “low tech” functions such as foregoing WhatsApp for SMS text messages and phone calls.

### Reflections on HCD

The work presented in this paper represents a first step in our co-design process, which has been followed by further work to refine solutions and develop implementation guidance for lay counselors and supervisors. It became clear that there was no singular solution that would be acceptable or feasible across all communities. Recognizing the importance of tailoring approaches to the distinct contexts in which each lay counselor group operates, our research approach shifted from developing specific “solutions” to which all lay counselors should adapt to presenting all possible solutions and facilitating lay counselors in identifying and prioritizing solutions that they felt would work best in their respective contexts. Noting recent critiques of design thinking as a form of colonialism [45] and the ethics of engaging in global health research as outsiders, we attempted to decenter ourselves as researchers and center the needs of the lay counselors and supervisors. This also influenced our decision to have supervisors lead the member-checking and other HCD activities that occurred in the later steps of our research.

To honor the voices of our participants, we felt it was important to present all the solutions. The solutions discussed included workarounds that the lay counselors or supervisors were already using; additional workarounds that they could use (although perhaps at considerable added cost or time to the counselors and supervisors); or outcomes that would better facilitate their work, although without a clear path or resources to achieve them. Many of the solutions generated by lay counselors and supervisors seemed to place the responsibility and burdens of addressing challenges on themselves. This may reflect a focus on short-term solutions that could be implemented with minimal resources and a resourcefulness developed from living in marginalized and underserved communities—another lasting impact of European settler colonialism. These solutions also highlight a limit of the co-design method used in this study: it supported sharing techniques and tips among participants in the room, but many solutions that could truly enhance their work required additional resources that neither they nor we were going to design our way out of needing. To equitably implement and sustain task-shifting models, especially when driven by US investment, resources must be allocated appropriately such that additional burdens (financial, logistical, and emotional) are not unduly placed on providers. This includes ensuring that research projects and support systems appropriately acknowledge and address barriers by providing financial support and resources to lay providers and other partners in low-resource settings.

### Reflections on Digital Health Equity

Given the tremendous gaps in access to mental health care worldwide, scalable and sustainable solutions are needed to increase access to care for the most underserved populations. There has been increased attention on the potential of digital tools such as mobile phone apps or internet-based treatments to address the mental health treatment gap by directly targeting clients and patients [46,47]. Despite the potential of these approaches, they may leave behind key groups that do not have access to cellphones or the ability to use them—thereby risking the creation or reinforcement of health inequities. In these instances, in-person treatment models may be necessary. Digital health tools can still play a key role in supporting in-person treatment delivery, particularly with lay counselors; however, considering the needs and preferences of lay counselors and supervisors while co-designing digital tools with them is essential in ensuring digital health equity. By allowing lay counselors and supervisors to suggest solutions to improve mobile phone supervision in our trial, we codeveloped multiple solutions with their needs and resources in mind. They not only generated solutions that could be used broadly but also, with intimate knowledge of their own settings, generated solutions that were uniquely suited to their experiences in their settings. These solutions may have the potential to increase equity in lay counselors’ access to mobile phones for supervision—with appropriate community-led adaptation based on the context—and, in turn, increase equity in access to mental health treatment in the communities in which they work.

### Limitations

These findings should be considered within the context of their limitations. The HCD approach used in this study allowed supervisors and lay counselors (ie, end users) to provide feedback and suggestions for the improvement of mobile phone supervision. However, as a result, our findings speak specifically to the use and optimization of mobile phones for lay counselor supervision in western Kenya. Although some findings may transfer to other settings or contexts, future work should aim to continually engage users across contexts and design and adapt solutions with these contexts in mind. Our number of supervisors for the study was also limited (n=3), which affects our ability to extrapolate from supervisor interviews but also underscores the importance of scalable and sustainable supervision. Similarly, 25% (6/24) of our lay counselors did not have smartphones, which affected their experience with supervision and qualitative responses. Finally, when appropriate, the interviews were translated, and all qualitative analyses were completed in English. In addition, the interviewers had ongoing relationships with supervisors and lay counselors, which may have affected reporting accuracy. Interview scripts and prompts were designed to investigate specific facilitators and barriers to remote supervision (eg, *What would make it easier for you to receive supervision by your mobile phone?*), and this wording may have influenced how supervisors and lay counselors responded as opposed to an unstructured interview format. Our coding team consisted of 1 native Kiswahili speaker who consulted the Swahili audio and answered team questions related to translation and coding for the Kiswahili interviews; however, it is possible that some nuances were lost in the translation to English.

### Conclusions

Task shifting offers an effective and potentially sustainable solution for closing the mental health treatment gap in low-resource settings; however, its scale-up and sustainment are limited by the need for ongoing supervision. Lay counselors and supervisors highlighted key benefits and challenges of using mobile phones and offered 27 distinct solutions to improve mobile phone supervision. Our findings underscore the benefits—and limitations—of co-designing solutions to improve the use of digital health tools and can serve as a foundation for future work that addresses barriers to the use of digital health tools in lower-resource settings.

## Appendix

Multimedia Appendix 1 Final interview guide.

Multimedia Appendix 2 COREQ (Consolidated Criteria for Reporting Qualitative Research) checklist.

Multimedia Appendix 3 Qualitative themes by use category.

## Abbreviations

BASIC: Building and Sustaining Interventions for Children
CHV: community health volunteer
COREQ: Consolidated Criteria for Reporting Qualitative Research
EBP: evidence-based psychotherapy
HCD: human-centered design
LMIC: low- to middle-income country
TF-CBT: trauma-focused cognitive behavioral therapy

## Data Availability

The data sets generated and analyzed during this study are available from the corresponding author upon reasonable request.
