# Supplementary material for: Understanding Lay Counselor Perspectives on Mobile Phone Supervision in Kenya: Qualitative Study
Source: JMIR Form Res. 2023 Feb 2;7:e38822. doi: 10.2196/38822 (PMC9936369; doi:10.2196/38822)
Supplement: Multimedia Appendix 1 [file formative_v7i1e38822_app1.docx]

**BASIC Semi-Structured Mobile Phone Supervision Qualitative Interview – Teacher Version**

INSTRUCTIONS FOR PARTICIPANT:

*Thank you for being willing to do an interview with us. We will be asking you some questions about your experience using mobile phones to communicate with your supervisor in your role as a Pamoja Tunaweza counselor. This interview will take about* ***one hour*** *to complete and will be* ***audio recorded for the purposes of transcription only and also not to miss out on any important information you give us****. Your answers will help us learn how to better support Pamoja Tunaweza delivery in schools. There are no right or wrong answers, we want to hear your honest opinions. Your individual responses are confidential and will not be shared with your Head Teacher, anyone from your school, your teacher coach, or the Ace Africa supervisors. Your answers will not impact any funding or resources you receive from your school or Ace Africa. Your responses will not be attached to your name or your school’s name. They will only be attached to a study ID number, and will only be viewed by the research team. Your responses will only be used to improve support for Pamoja Tunaweza. As a participant, you will receive 500 ksh for completing this interview.*

*For this interview,* ***we are interested in learning about your experience using mobile phones to communicate with your supervisor in your role as a Pamoja Tunaweza counselor.*** *When we talk about communicating with your supervisor using mobile phones, we mean any way you have communicated with supervisor in relation to Pamoja Tunaweza supervision. This may include phone calls, SMS, WhatsApp messages, or any other way of communicating with your supervisor with your phone about Pamoja Tunaweza supervision. Please think about* ***all*** *these activities when you’re answering questions about mobile phone supervision. We will be asking about what you liked about communicating with your PT supervisor through your mobile phone, the challenges you faced, and what could be improved.*

***Interviewer note:*** *Throughout the interview, please remind the participant not to mention any specific names but to specify roles (e.g., Head Teacher, Deputy Teacher, Senior Teacher, Coach, Supervisor, ACE staff).*


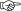


**1. Please think about the entire time you have been a Pamoja Tunaweza counselor. Tell me about your experience using mobile phones to communicate with your supervisor in your role as a Pamoja Tunaweza counselor.**

**2. What do you like most about communicating with your Pamoja Tunaweza supervisor through your mobile phone about Pamoja Tunaweza supervision?**

**3. What is challenging or frustrating about communicating with your Pamoja Tunaweza supervisor over your mobile phone?**

***Follow-up question:* How did you overcome those challenges?**

**4. How possible do you think it would be to replace your current in-person supervision with supervision through mobile phones? We’ll use a scale of 1-10, with 1 being not at all possible and 10 being extremely possible.**

*Follow-up question:* You’ve just given me a rating of [the rating]. Tell me more about why you chose that?

**5. What situations would you need face-to-face supervision and would not want that to be replaced by mobile phone supervision?**

**6. What would make it easier for you to receive supervision by your mobile phone?**

**7. How satisfied would you be replacing in-person supervision with supervision using mobile phones? We’ll use a scale of 1-10, where 1 is not at all satisfied and 10 is extremely satisfied.**

*Follow-up question:* You’ve just given me a rating of [the rating]. Tell me more about why you chose that?

**8. What would make you more satisfied with receiving supervision over your mobile phone?**

**9. Imagine you are preparing for a Pamoja Tunaweza group or individual visit and need support with a specific skill or step sheet for a visit. Please tell me like a story, step-by-step, how you would contact your supervisor to receive support over your phone?**

**10. Is there anything else about receiving supervision by your mobile phone that I haven’t asked about that you’d like to share?**
