# Supplementary material for: Understanding Lay Counselor Perspectives on Mobile Phone Supervision in Kenya: Qualitative Study
Source: JMIR Form Res. 2023 Feb 2;7:e38822. doi: 10.2196/38822 (PMC9936369; doi:10.2196/38822)
Supplement: Multimedia Appendix 3 [file formative_v7i1e38822_app3.docx]

Percentage of interviews in which acceptability themes appear, by usage category.

| Theme | | High Frequency (N=8) | Average Frequency (N=9) | Low Frequency (N=7) | Supervisors (N=3) |
| --- | --- | --- | --- | --- | --- |
|  | |  |  |  |  |
| **Likes** | | **8 (100%)** | **9 (100%)** | **7 (100%)** | **3 (100%)** |
|  | Decreasing Burden | 7 (88%) | 9 (100%) | 4 (57%) | 3 (100%) |
|  | Facilitating Support | 8 (100%) | 9 (100%) | 7 (100%) | 3 (100%) |
|  | Increasing Independence | 2 (25%) | 2 (22%) | 0 (0%) | 0 (0%) |
| **Dislikes** | | **5 (63%)** | **5 (56%)** | **3 (43%)** | **3 (100%)** |
|  | Limited information transmission | 2 (25%) | 3 (33%) | 3 (43%) | 3 (100%) |
|  | Limited relationship with supervisors | 2 (25%) | 3 (33%) | 0 (0%) | 0 (0%) |

Percentage of interviews in which feasibility themes appear, by usage category.

| Theme | | High Frequency (N=8) | Average Frequency (N=9) | Low Frequency (N=7) | Supervisors (N=3) |
| --- | --- | --- | --- | --- | --- |
|  | |  |  |  |  |
| **Facilitators** | | **8 (100%)** | **9 (100%)** | **7 (100%)** | **3 (100%)** |
|  | Access to working smart phones | 7 (88%) | 9 (100%) | 7 (100%) | 2 (67%) |
|  | Ease and convenience of mobile supervision | 6 (75%) | 4 (44%) | 3 (43%) | 2 (67%) |
|  | Supervisor-counselor relationship | 3 (38%) | 1 (11%) | 1 (14%) | 0 (0%) |
|  | Mobile phone literacy | 2 (25%) | 3 (33%) | 3 (43%) | 1 (33%) |
| **Barriers** | | **8 (100%)** | **9 (100%)** | **7 (100%)** | **3 (100%)** |
|  | Limited Resources and Time | 6 (75%) | 7 (78%) | 6 (86%) | 3 (100%) |
|  | Technical Barriers/Difficulties | 8 (100%) | 9 (100%) | 6 (86%) | 3 (100%) |
|  | Communication Challenges | 8 (100%) | 9 (100%) | 5 (71%) | 3 (100%) |
|  | Limitations on Activities | 7 (88%) | 6 (67%) | 5 (71%) | 3 (100%) |
